# Editorial: Bone aging and osteoporosis: recent evidence focusing on plant-based natural products

**DOI:** 10.3389/fmed.2024.1384493

**Published:** 2024-03-01

**Authors:** Ling-Feng Zeng, Claudio Lucchiari

**Affiliations:** ^1^State Key Laboratory of Traditional Chinese Medicine Syndrome, The Second Affiliated Hospital of Guangzhou University of Chinese Medicine, Guangzhou, China; ^2^Guangdong Provincial Key Laboratory of Chinese Medicine for Prevention and Treatment of Refractory Chronic Diseases, Guangdong Provincial Hospital of Chinese Medicine, Guangzhou, China; ^3^Department of Philosophy, University of Milan, Milan, Italy

**Keywords:** plant-based natural products, bone aging, osteoporosis, evidence, mechanisms

Various active ingredients from plants, such as polyphenols, polysaccharides, and flavonoids, are considered potential sources of drugs for treating bone aging and osteoporosis (OP) and have been proven to have antioxidant, anti-inflammatory, and anti-bone resorption effects; thus, these compounds alleviate bone aging and OP (1, 2). However, unfortunately, studies on many active ingredients from plants with therapeutic potential for OP are still in the laboratory stage rather than being applied in clinical practice. Therefore, we proposed to summarize the research on this Research Topic to promote further discovery and research on plant natural products, and to promote the clinical application of plant-derived natural products. A total of 13 studies were included in this review, and these studies mainly presented the research progress and development of single plant medicines, monomeric compositions, and Chinese patent medicines for the treatment of bone aging-related diseases. The types of articles on this topic were mainly reviews, which can help us better understand the widespread progress of plant-derived natural products in the treatment of bone aging. In addition, we propose that more attention should be given to clinical research and mechanistic explorations of natural plant products.

## Research on the mechanism of plant-based natural products

Basic research is key for promoting the application of natural plant products in the clinical treatment of bone aging and OP. A total of three basic research studies will be discussed. Jie et al. identified palmatine (PAL) as the main active ingredient of ErXian decoction by using liquid chromatography-tandem mass spectrometry and network pharmacology screening and explored the therapeutic effect of PAL in the treatment of OP and osteoarthritis (OA) comorbidities in rats. Their results indicated that PAL can significantly improve the femoral microstructure and alleviate cartilage damage in OA-OP rats, and its mechanism of action may regulate the gut microbiome and serum metabolites to alleviate bone aging in OA-OP patients. Aconine has anti-inflammatory and analgesic pharmacological effects, but it must be used with caution due to its toxicity, and this limitation needs to be emphasized. Xue et al. reported that aconine can effectively reduce vertebral bone loss and restore the high level of bone turnover markers in ovariectomized mice. These authors further confirmed that aconitine can reduce the expression of the osteoclast-specific genes NFATc1, c-Fos, Cathepsin K, and Mmp9, thereby significantly inhibiting the occurrence of osteoclasts. The mechanism of action of aconitine may be related to the inhibition of NF-κB signaling pathway-mediated ferroptosis and the formation of osteoclasts. Liang et al. discovered through metabolomics techniques that Longbie capsules can regulate serum lipid metabolism, amino acid metabolism, and estrogen levels, thereby helping maintain the balance in bone metabolism in OA-OP rats and reducing bone loss in the articular cartilage. The research of Liang et al. provides a replicable strategy for elucidating the mechanism of traditional Chinese medicines in treating bone aging diseases.

## Evidence-based therapeutic applications of plant-derived natural products

Evidence-based medicine is an indispensable part of clinical decision-making. The application of natural plant products from the laboratory into clinical practice requires a large amount of evidence-based data. Zhao et al. used quantitative data analysis methods to evaluate the efficacy of resveratrol in treating OP model rats. This meta-analysis included 15 animal experiments and evaluated multiple indicators, including bone density and serum marker levels. In this study, they found that resveratrol can increase bone density, improve bone microstructure, and regulate calcium and phosphorus metabolism in OP model rats. Resveratrol is a natural polyphenolic compound that has been proven to have anti-inflammatory, antioxidant, and anti-aging effects in multiple pharmacological studies (3, 4). Undoubtedly, Zhao et al.'s research provides reliable evidence for the use of resveratrol in the treatment of OP, which will promote its clinical application. Osteoporosis compression fracture, which is a common complication of OP, is a substantial economic and medical burden. The prevention and treatment of osteoporotic fractures cannot be ignored in public health. Fu et al. conducted a meta-analysis and confirmed that the Jintiange capsule has good safety and efficacy in the treatment of osteoporotic vertebral compression fractures (OVCFs). Specifically, Jintiange capsules can increase bone density, alleviate pain, and reduce the incidence of adverse events. The evidence provided by Fu et al. will further promote the clinical application of Jintiange capsules in the treatment of OVCFs.

## Comprehensive review of plant natural products

A total of eight descriptive reviews will be summarized to help readers comprehensively understand the current state of the application of plant-based natural products to treat bone aging and OP. After analyzing and summarizing the pathological mechanisms of OP, Zhou C. et al. focused on the effects of traditional Chinese medicine formulas and their chemically active components on osteoblasts, osteoclasts, bone marrow mesenchymal stem cells, bone microstructure, angiogenesis, and the immune system; these findings provide a good perspective for comprehensively understanding of the mechanism of action of classical Chinese medicine formulas in the treatment of OP. Zhou G. et al. summarized the mechanism of action of traditional Chinese medicine formulas and monomers in the treatment of KOA combined with OP; these results will help readers understand the role of traditional Chinese medicine in the treatment of KOA combined with OP. During the aging process, cellular aging is an instinctive response of cells to various exogenous and endogenous stimuli. Therefore, targeting cellular aging may be a potential strategy for treating OP. Zhang et al. summarized the potential of traditional Chinese medicine in the treatment of senile OP (SOP) from the perspective of cell aging. Based on the current research, Zhang et al. suggested that the use of traditional Chinese medicine formulations and their active ingredients for targeting cellular aging in the treatment of SOP has broad application prospects. The potential of plant natural products to target cellular aging and exert anti-OP therapeutic effects is worthy of further exploration and research. Tang et al. comprehensively summarized the potential of *Cornus officinalis* for treating OP, and they also summarized the effective active ingredients of *Cornus officinalis*; this study provided a complete list of possible effective ingredients in *Cornus officinalis* for the treatment of OP. Wang et al. reviewed the impact of the pilose antler polypeptide on the mechanism of bone homeostasis; these results provided detailed evidence of the role of the pilose antler polypeptide in maintaining the dynamic balance of osteoblasts and osteoclasts. Gu et al. summarized research on the treatment of rheumatoid arthritis (RA) with OP using single traditional Chinese medicine formulations. Similar to the studies of Zhou G. et al., Gu et al.'s study provides additional information on the use of traditional Chinese medicine for the treatment of the comorbidity of RA combined with OP. Tea, as a leisure drink, is loved by many people worldwide. Xie et al. summarized the mechanism of tea in treating OP, OA, and RA, and the results provide comprehensive information on the use of tea in treating these three diseases related to bone aging. Zeng et al. offered significant research advancements that can be utilized as guidelines for the treatment of KOA with integrative medicine based on traditional Chinese medicine. The publication of guidelines will promote the standardization of clinical practice. We hope that these guidelines can provide scientific and effective guidance for the treatment of KOA and that the herbal therapies included in these guidelines will be thoroughly validated in a wider range of clinical applications.

In summary, multiple studies have investigated the use of plant-based natural products for the treatment of bone aging and OP. These studies provide convenient and extensive information on the use of plant-based natural products, especially traditional Chinese medicines, in the treatment of diseases associated with aging bone. We thank all the authors, peer reviewers, and Frontiers staff for their rigorous and professional contributions. Without their help, successfully presenting this summary would not have been possible. We believe that all the information contained in this review will be beneficial for promoting scientific progress in the study of plant-based natural products for bone aging.

## Author contributions

L-FZ: Conceptualization, Writing – original draft, Writing – review & editing. CL: Writing – review & editing.
